# Commentary on my personal experience of patient and public involvement in the TOPSY trial

**DOI:** 10.1186/s13063-023-07254-8

**Published:** 2023-03-25

**Authors:** Margaret Graham, Kirsteen Goodman

**Affiliations:** 1 PPI representative, Dunlop, Ayrshire, UK; 2 NMAHP Research Unit, Glasgow Caledonian University, Glasgow, UK

**Keywords:** PPI, RCT, Lessons learned

## Background to commentary

Patient and public involvement (PPI) in research is now widely expected and encouraged at all stages of the research cycle. PPI members provide expert knowledge of the lived experience and their contribution is crucial. The plethora of publications on PPI has increased almost threefold in the 5-year period from 2017 to 2022 (37,136 results) compared to the preceding 5-year period 2012 to 2017 (12,528 results) (PUBMed search for terms “Patient and Public Involvement”). Many of these publications focus on PPI impact or frameworks, but few focus on the personal experiences of the patient and public representative journey.

In the TOPSY trial (The Treatment Of Prolapse with Self-care Pessary), one patient and public representative, Margaret Graham, started her journey as a patient and public representative at the grant writing stage as a co-applicant in 2017. The TOPSY trial is now in a long term follow-up stage.

The aim of this publication is to highlight, via a personal experience biography, the personal journey that Margaret has taken in her role as a patient and public representative. This account, written by Margaret, highlights the mutual learning between her and the research team. This mutual learning experience included discussions around areas where the study team could have done better but also highlights ideas that did work well. We also hope this will encourage more patient and public representatives to write about their journey to enhance the research communities’ knowledge sharing on this very important role in the research team and to ultimately make being involved in research the best experience it can be.

## PPI personal experience biography

In the summer of 2017, I filled in an expression of interest form offered to me by the nurse in the Gynaecology clinic. I had been to see her to have my pessary checked and to make sure that no problems had developed since my last visit 6 months before.

It had been a great relief a couple of years previously when the doctor in the clinic suggested that a pessary could help with my prolapse problems after a referral by my GP. The idea of a more invasive type of help like surgery had not been an appealing idea. Therefore, when I started using a ring pessary and found it worked well, I was really happy. With occasional checks and support by the nurses in the clinic, I was much more comfortable day to day. Laterally, the nurse (who I got to know a little) had also encouraged me to insert and remove the pessary myself at home if it suited me. I had been timid about this at first since I worried that I might do internal damage or if I did not have a regular visit to the clinic perhaps it all might become irritated. But with her reassuring me in the clinic and practice at home, I had become confident and managed the pessary quite well.

A short time after filling out and sending off the notification of interest for a study about pessary use, I was contacted by Carol Bugge who then came to interview me and talk about my experiences using a pessary and other questions relating to my healthcare. Additionally, we touched base on NHS care for British women and their experience of female specific healthcare which, as mother to three young women, I do wish was more comprehensive.

Carol talked about a proposed study into effective methods of self-managed pessary usage and how it might improve patients’ quality of life. She explained how patient representation was important and indeed expected. Since I had been already predisposed to favour the idea of women having more autonomy within their care route, the idea of the study made sense and I was stimulated to become involved in the preliminary work before the trial proposal to the National Institute for Health and Care Research (NIHR).

Gradually over the next few months, some aspects of my role as a PPI representative became a little clearer with lots of guidance by Carol and then also the trial manager. At this point, the question of some training by a group like INVOLVE^1^ was mentioned, and with hindsight, I realise I should have pursued this to back-fill the beginner aspects of my slightly sketchy grasp of what to expect and even the structure of clinical trials. I nevertheless started contributing by giving feedback on proposed patient-facing leaflets, questionnaires and letters, trying to consider the point of view of a potential TOPSY woman coming into the study. I also attended meetings on the telephone with the original trial team and groups of clinicians, other health professionals such as nurses, physiotherapists and also statisticians and technology designers.

It was suggested that I and another PPI become co-applicants and members of the Project Management Group. Eventually, once the protocol was accepted by NIHR and was needing co-applicant signatures, I was taken aback to find myself in a list with the status of an institution like a hospital or university. In fact, the signing process was pretty intimidating mainly because of the online routines involved, but the trial organisers had anticipated this and walked me through the more complex procedures. Still, the legal wording did give me pause for thought; what responsibility had I legally? I felt a bit out of my depth briefly.

Eventually, at the end of 2017, it was a revelation to attend a large face-to-face meeting at Caledonian University and see a lot of the people involved in TOPSY in person. At this meeting, explanations around some of the process of setting up the database and a very interesting series of discussions about statistical aspects of trials and clinical considerations left me feeling stimulated though challenged my intellect—I had to reassure myself that my role as an end user or patient-under-treatment was exactly why I was needed there. During this whole time, I tried to take in the very alien world of preparation for a clinical trial. It always proved very interesting though often quite confusing and sometimes even funny—the group trying to come up with a suitable trial name passing on OOPSY and finally deciding on TOPSY or Treatment Of Prolapse with Self-care Pessary.

Over that period to spring 2018 when the first patient was randomised (new vocabulary for me), I was surprised to realise that I was excited for the trial to start with its embedded pilot study which provided really interesting feedback.

Over that year into 2019, I was then involved in the Process Evaluation study protocol and that year I became a member of the qualitative committee which was blinded and I met the academic researcher, who has been so supportive of my role carefully explaining really fresh (to me) concepts relating to her work. The qualitative analysis is reliant on translation of the interviews with randomised and non-randomised women and the health professionals involved and the statistical models and tables feed information for consideration by the committee and later to the study as a whole. Some of this is difficult to understand but I found the small committee set up very approachable when I really had questions about procedure or just science! I had always found the trial lead easy to ask questions of and in fact she had encouraged me to contact her with any problems. Later on, she acted as a bridge between me and the University of Stirling payment system which failed me on occasion, and then the academic researcher also helped with this very willingly.

I have felt a bit nonplussed at times by the lacks in the payroll system which Carol worked to sort and apologised for so fully. The problem has been my status as a non-member of the university staff and also my intermittent accessing of the university database. In the last year to 2021, this has been resolved by changing my status to a temporary casual research fellow with, importantly, a yearly contract. Now, I can even access my own payslips.

Importantly for me my involvement in TOPSY meetings has been mainly by teleconference or by Microsoft Teams, which means that I can continue in my main job as a full-time carer for my husband in our home. Then, during the pandemic lockdowns, the setup was easy to navigate since virtual meetings were already established.

An interesting development is that in the last year or so, the four PPIs involved meet regularly usually with the trial manager and with a clinician or perhaps an academic present too. I think initially these catch-ups were seen as social and supportive and were un-minuted. Indeed, it proved so for me anyhow and I enjoyed the chance to remember that all these leaflets and models and numbers referred to people like me and these other women. Lately, the tone became more serious with one PPI explaining her situation with initial involvement in the oversight group for the mesh report by Baroness Cumberlege and then ensuing work by NHS England and then the links with so many areas not least the TOPSY trial.

The novelty of working for the trial and the rigour therein has been a fascinating and rewarding experience. The novelty of learning about new areas and disciplines was made all the more enjoyable by the friendly and welcoming attitude of many of the professionals involved who also provided me with structured or impromptu support.

This help went some way to mitigate the more difficult aspects of being involved in this trial: my rather opaque status; the slightly perplexing issues relating to my confusion about various aspects of procedure or protocol; my uncertain grasp of how pro-active a PPI role was expected to be, because of no real introductory, preparatory training or information relating to the role of PPI in a NIHR Trial; problems in accessing the payment system until I became a temporary research fellow. Meeting other PPIs and initial training might have given support and potentially some type of formalised, beginner template might have outlined useful activities like diary-keeping. Sometimes, it was hard to quell a touch of ‘imposter syndrome’ in meetings where the language was technical or too many mnemonics were reeled off quickly. Indeed, even the complexity of the structure and methods of a trial were quite hard to understand. In many ways, it was just a case of realising what not to concern myself with.

On the other hand, the role of PPI on the TOPSY Trial was easily assimilated into my normal work routine as a home based full-time carer. The trial used TEAMS and teleconferences as a major mode of communication with only occasional face to face meetings. Certainly, this system segued smoothly into the online routines relating to the COVID-19 pandemic, the only detrimental aspect being my very creaky home computing and printing setup. I had a tremendous feeling of genuine involvement and after a period of adjustment felt that I had a useful position in TOPSY. I enjoyed interactions with the many and varied professionals and more recently contact with and the company of other PPI members and the trial manager who has provided an encouraging and supportive online meeting environment for a more informal ‘catch up’. Overall, I feel hopeful that I have been able to feed back some value to the NHS while making a contribution to consistent whole life healthcare for women.

## Data Availability

N/A for this commentary.

## Footnotes

1 INVOLVE was dissolved in 2020 and was subsequently incorporated into the NIHR Centre for Engagement and Dissemination which includes Be Part of Research, which encourages patients to participate in clinical research https://bepartofresearch.nihr.ac.uk. However, INVOLVE was the national advisory body at the time and this was the information given to Margaret.

